# Knockdown of HE4 suppresses aggressive cell growth and malignant progression of ovarian cancer by inhibiting the JAK/STAT3 pathway

**DOI:** 10.1242/bio.043570

**Published:** 2019-09-15

**Authors:** Aihong Wang, Canhui Jin, Xiaoyu Tian, Ying Wang, Hongyu Li

**Affiliations:** 1 Department of Gynecologic and Obstetrics, The First Affiliated Hospital, College of Clinical Medicine, Henan University of Science and Technology, Luoyang 471000, Henan Province, China; 2 Department of Gastrointestinal Tumor Surgery, The First Affiliated Hospital, College of Clinical Medicine, Henan University of Science and Technology, Luoyang 471000, Henan Province, China; 3 Department of Gynecologic Oncology, The Third Affiliated Hospital of Zhengzhou University, Zhengzhou 450052, Henan Province, China

**Keywords:** Ovarian cancer, HE4, Cell growth, Malignant progression, JAK/STAT3 pathway

## Abstract

Human epididymis protein 4 (HE4) is well known to be a predictor of ovarian cancer clinically. HE4 is reported to play crucial roles in ovarian cancer progression and metastasis. The purpose of the present study was to explore its biological role and molecular mechanism in ovarian cancer. In our study, we found that expression levels of HE4 in tissues, serum and urine in ovarian cancer were upregulated compared to healthy and benign groups. HE4 expression was elevated in ovarian cancer cells. Knockdown of HE4 dampened cell proliferation and Ki67 expression, as well as enhanced apoptosis, caspase-3 activity and cleaved-caspase-3 expression. In addition, HE4 downregulation repressed invasion and migration capabilities of ovarian cancer cells. Western blot analyses showed that knockdown of HE4 reduced the levels of matrix metalloproteinases (MMP-2 and MMP-9) and inhibited epithelial to mesenchymal transition (EMT) in ovarian cancer cells. *In vivo* animal experiments revealed that HE4 downregulation constrained the growth of xenograft tumor. Mechanism research showed that knockdown HE4 inhibited the activity of JAK/STAT3 pathway *in vitro* and *in vivo*. In conclusion, our findings reported that knockdown of HE4 suppresses aggressive cell growth and malignant progression of ovarian cancer by inhibiting the JAK/STAT3 pathway, which provides valuable insights to contribute to develop novel HE4-targeted therapies.

## Abstract (precis)

**Summary:** Our findings reported that HE4 knockdown suppresses aggressive cell growth and malignant progression of ovarian cancer by inhibiting the JAK/STAT3 pathway, which could provide a valuable insight into developing novel HE4-targeted therapies.

## INTRODUCTION

Ovarian cancer arises in the ovary, and is the most lethal gynecological malignancy with a high mortality rate (45%), which features aberrant cell growth and peritoneal disseminated metastasis (Zhang et al., 2018). As the disease is asymptomatic at early stages, most cases are generally detected at an advanced stage and thus seriously affects the survival rate of patients (James et al., 2018). In spite of great progress in chemotherapy and surgical therapies for the disease, the majority of patients still develop relapse or metastasis, and the 5-year survival rate of patients who are diagnosed as having peritoneal metastasis is about 30% (Vaughan et al., 2011). Zhang and his colleagues have reported that invasion and metastasis of ovarian cancer at early stage are the leading reasons for its high mortality and poor prognosis (Zhang et al., 2014). It is well known that tumor progression is coordinated by complicated interactions between tumor cells and their microenvironment (Gomes et al., 2018). Consequently, it is of great significance to explicate the molecular mechanism of the pathogenesis of ovarian cancer for early diagnosis and treatment in order to improve the prognosis of patients.

Human Epididymis Protein 4 (HE4), a putative protease inhibitor, also is known as WAP four-disulfide core domain protein 2 and encoded by the *WFDC2* gene (Hellstrom et al., 2003; Li et al., 2013). HE4 has been reported to be a tumor marker of ovarian cancer with 80% sensitivity at a cut-off 150 pmol/L (Molina et al., 2011; Zhu et al., 2016). Previous studies of HE4 have emphasized the likely clinical application of HE4 as a biomarker and predictor. For example, previous published studies have shown that serum HE4 concentrations are significantly upregulated in ovarian cancer patients compared with patients with benign disease or healthy controls (Hamed et al., 2013), and combining HE4, CA125 and age as a diagnostic may optimize referral and diagnosis of patients with suspected ovarian cancer (Karlsen et al., 2015). Nonetheless, most studies in to the roles of HE4 in the malignant biological behaviors of ovarian cancer are in dispute. Research has reported that HE4 can promote the proliferation, invasion and metastasis of ovarian cancer cells (Zhuang et al., 2014; Zhu et al., 2016). An opposite result presented by Kong et al. has shown that HE4 might repress cell proliferation by regulation of the MAPK/PI3K/AKT pathways and then serve a protective function in ovarian cancer progression (Kong et al., 2014). Recently, mounting research has focused on the roles of molecularly targeted agents in anti-cancer activity for patient. Little is known about the HE4 related molecular alterations and its mechanism in the malignant phenotype of ovarian cancer.

Herein, our aims are to investigate the profile of HE4 expression in tissue, serum, urine and cells of ovarian cancer and the effects of HE4 knockdown on malignant biological behaviors of cells as well as its molecular mechanisms. Our findings might contribute to developing a new and effective molecularly targeted agent for ovarian cancer therapy.

## RESULTS

### Expression profiles of HE4 in tissues, serum and urine in ovarian cancer

**Fig. 1.**
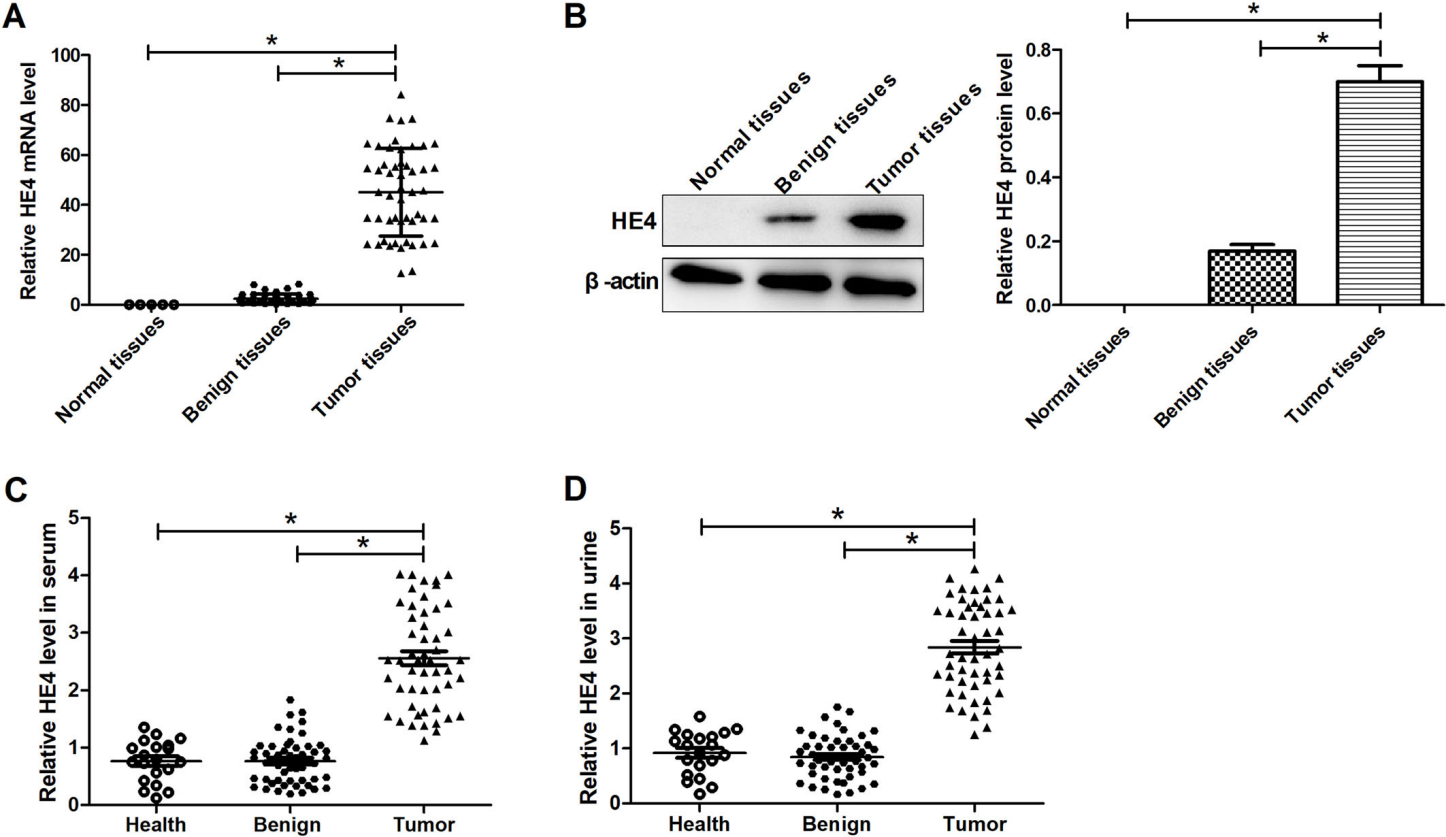
**Expression profiles of HE4 in tissues, serum and urine in ovarian cancer.** (A) qRT-PCR determination of the expression levels of HE4 in 51 ovarian cancer, 50 benign ovarian cancer and normal tissue samples. (B) Western blot analyses of the expression levels of HE4 in ovarian cancer, benign ovarian cancer and normal tissue samples. ELISA detection of 51 ovarian cancer, 50 patients with benign ovarian cancer and 20 healthy serum (C) and urine samples (D). *P<0.05.

To explore the effects of HE4 expression on the malignant progression of ovarian cancer, the expression levels of HE4 were detected in 51 ovarian cancer samples, 50 benign ovarian cancer samples and five normal tissue samples, 51 ovarian cancer, 50 patients with benign ovarian cancer and 20 healthy serum and urine samples. The results from qRT-PCR and western blot analyses showed that HE4 expression levels in tissue, serum and urine of ovarian cancer patients were significantly upregulated compared with normal or benign groups (Fig. 1A–D).

### HE4 expression was increased in ovarian cancer cells

**Fig. 2.**
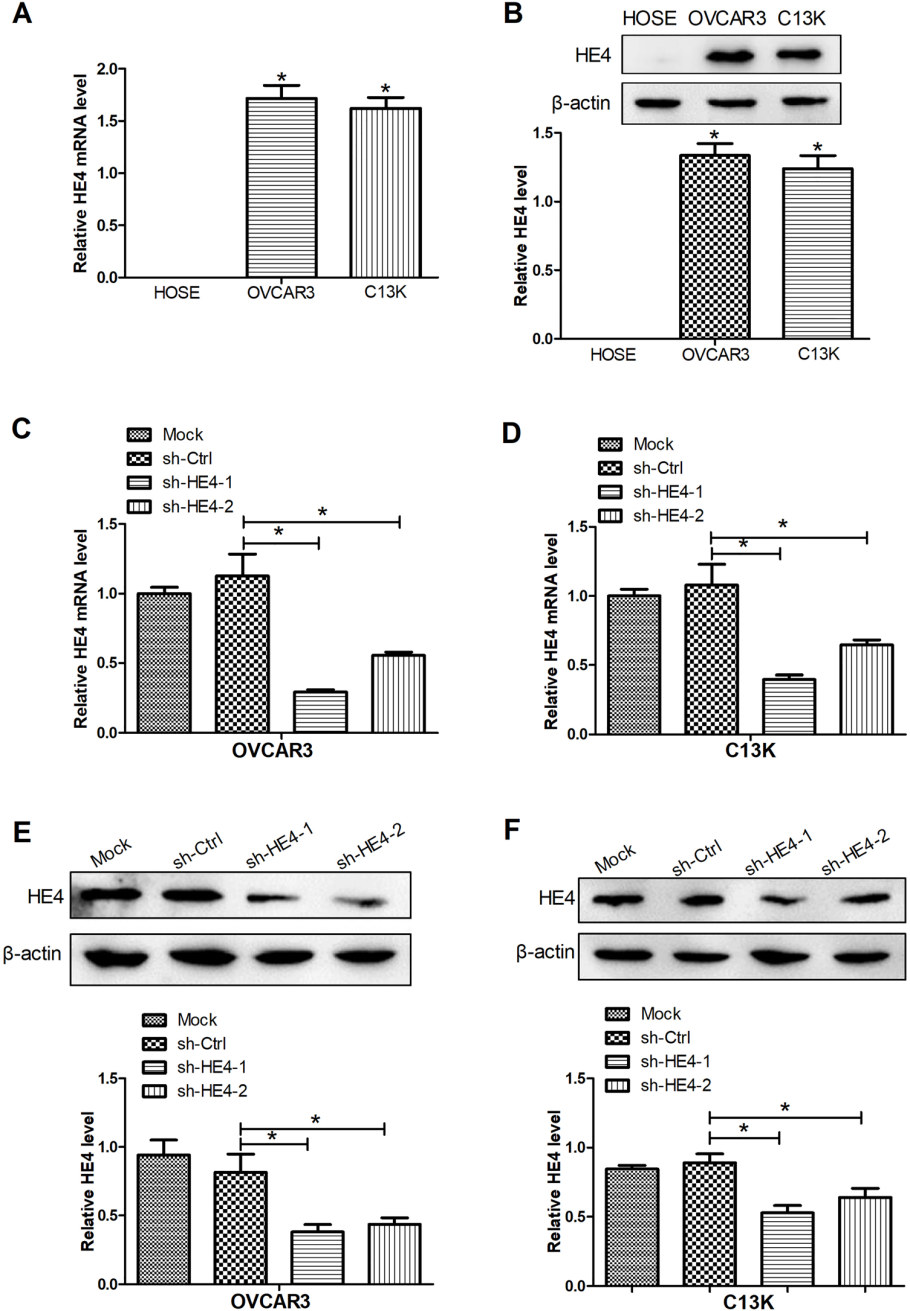
**Expression of HE4 in ovarian cancer cells.** mRNA (A) and protein (B) expression levels of HE4 were detected by qRT-PCR and western blot assays, respectively. qRT-PCR was used to evaluate the levels of HE4 in OVCAR3 (C) and C13K (D) cells transfected with sh-HE4-1, sh-HE4-2, or sh-Ctrl. Western blot assays were conducted to measure the protein levels HE4 in transfected OVCAR3 (E) and C13K (F) cells. *P<0.05.

OVCAR3 and C13K cell lines were appropriate model systems in which to study initiation and progression of ovarian cancer. In our study, the HE4 expression was measured by qRT-PCR and western blot assays. The results showed that HE4 expression was not detected in HOSE cells and a significant rise in HE4 mRNA and protein expression was observed in OVCAR3 and C13K cells compared with HOSE cells (Fig. 2A,B). To further investigate the roles of HE4 in ovarian cancer cells, knockdown of HE4 was performed by stable transfection of shRNAs targeting HE4 (sh-HE4-1 and sh-HE4-2) into cells. The results of HE4 knockdown efficiency showed that the mRNA and protein expressions of HE4 were obviously downregulated in OVCAR3 and C13K cells transfected with sh-HE4-1 and sh-HE4-2, compared with the non-transfected cells (Fig. 2C–F). In addition, sh-HE4-1 exerted stronger knockdown efficiency compared to sh-HE4-2 in OVCAR3 and C13K cells. Therefore, sh-HE4-1 (sh-HE4) was chosen for the further experiments.

### Downregulation of HE4 suppressed cell proliferation and promoted apoptosis in ovarian cancer cells

**Fig. 3.**
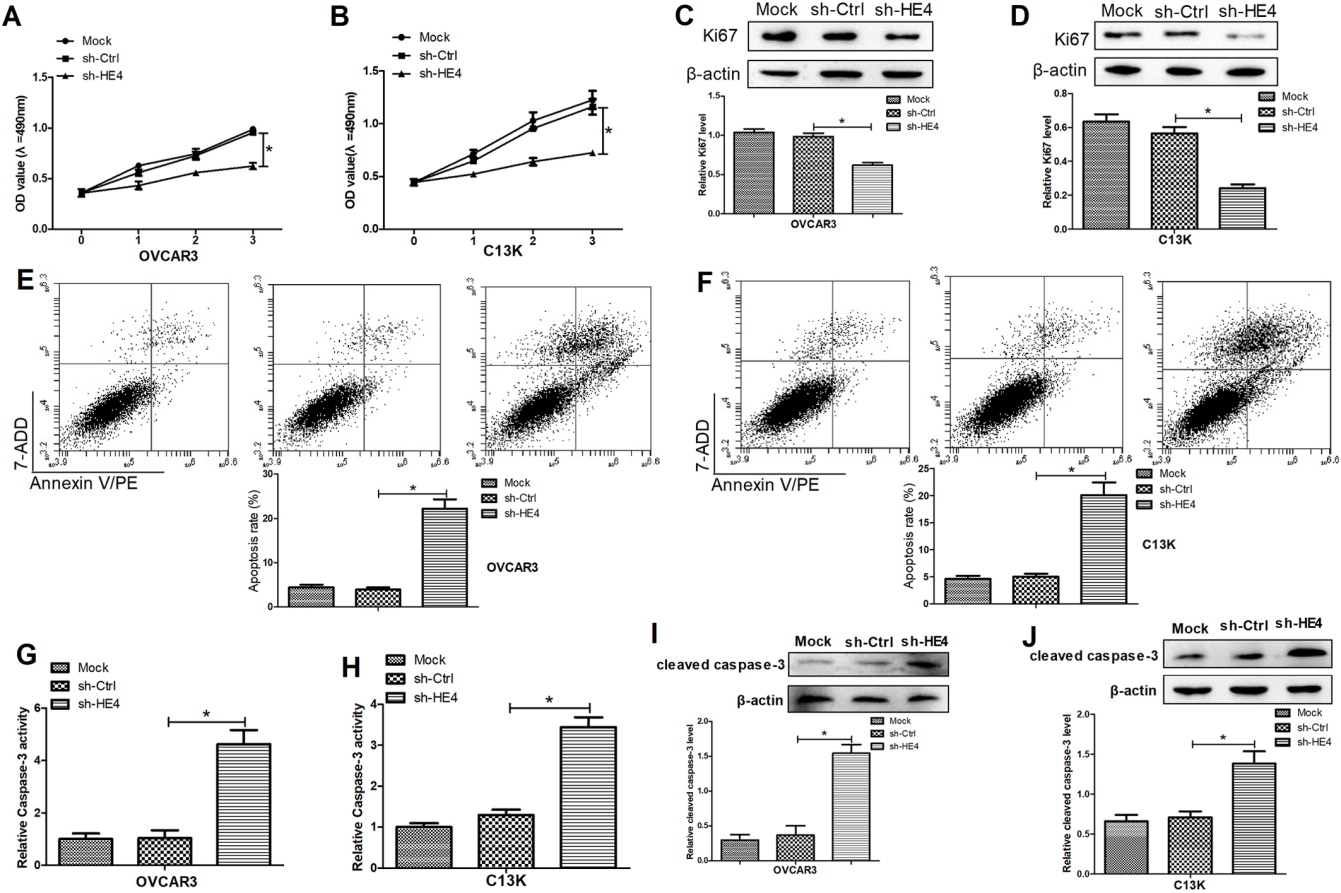
**Downregulation of HE4 suppressed cell proliferation and promoted apoptosis in ovarian cancer cells.** MTT assays were performed to determine the proliferation of OVCAR3 (A) and C13K (B) cells transfected with sh-HE4 or sh-Ctrl. Western blot assays were conducted to detect the expression of Ki67 in the transfected OVCAR3 (C) and C13K (D) cells. Flow cytometry assays were carried out to measure the apoptosis in the transfected OVCAR3 (E) and C13K (F) cells. ELISA assays were used to detect caspase-3 activity in culture supernatants of the transfected OVCAR3 (G) and C13K (H) cells. The expression of cleaved caspase-3 in the transfected OVCAR3 (I) and C13K (J) cells was determined by western blot assays. *P<0.05.

In order to investigate the roles of HE4 in cell proliferation and apoptosis in OVCAR3 and C13K cells, MTT assays and flow cytometry analyses were conducted in the cells transfected with sh-HE4. The results from MTT assays uncovered that HE4 knockdown significantly inhibited proliferation of OVCAR3 and C13K cells (Fig. 3A,B). Furthermore, an obvious drop in expression of Ki67, a proliferation mark protein, was determined in HE4-knockdown OVCAR3 and C13K cells compared with the control group (Fig. 3C,D). In addition, flow cytometry analyses showed that HE4 downregulation significantly induced the apoptosis in OVCAR3 and C13K cells transfected with sh-HE4 compared with the control group (Fig. 3E,F). Moreover, the increased caspase-3 activity was determined in culture supernatants of OVCAR3 and C13K cells transfected with sh-HE4 and (Fig. 3G,H), and the expression of cleaved caspase-3 in the transfected cells was also significantly constrained (Fig. 3I,J). These results suggested that HE4 downregulation suppressed cell proliferation and promoted apoptosis in ovarian cancer cells.

### Downregulation of HE4 repressed cell invasion and migration of ovarian cancer cells

**Fig. 4.**
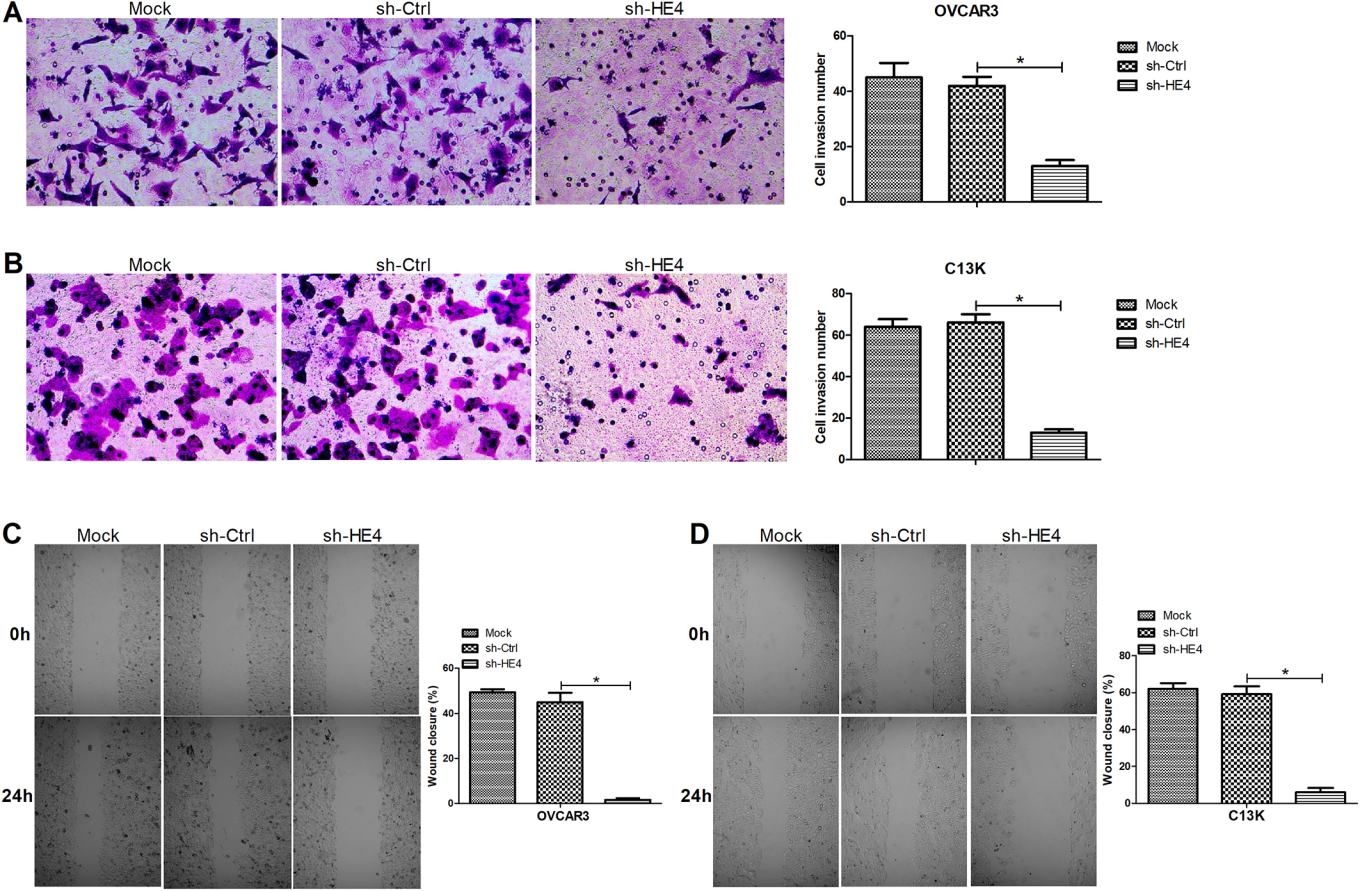
**Downregulation of HE4 repressed cell invasion and migration of ovarian cancer cells.** Transwell assays were carried out to evaluate the invasion of OVCAR3 (A) and C13K (B) cells transfected with sh-HE4 or sh-Ctrl, at 48 h, amplification, ×200. Wound healing assays were performed to determine the migration of the transfected OVCAR3 (C) and C13K (D) cells at 0 and 24 h, amplification, ×40. *P<0.05.

Further, transwell and wound healing assays were carried out to evaluate the effects of HE4 on invasion and migration capabilities of OVCAR3 and C13K cells. As shown in Fig. 4A and B, HE4 knockdown markedly dampened the invasion capability of the transfected OVCAR3 and C13K cells compared with the control group. Moreover, the results obtained from wound healing assays revealed that there was a significant decline in the migration ability of OVCAR3 and C13K cells transfected with sh-HE4, compared with the control group (Fig. 4C,D). These data concluded that HE4 downregulation inhibited cell invasion and migration of ovarian cancer cells.

### Downregulation of HE4 inhibited MMPs expression, mesenchymal transition (EMT) and the JAK/STAT3 pathway in ovarian cancer cells

**Fig. 5.**
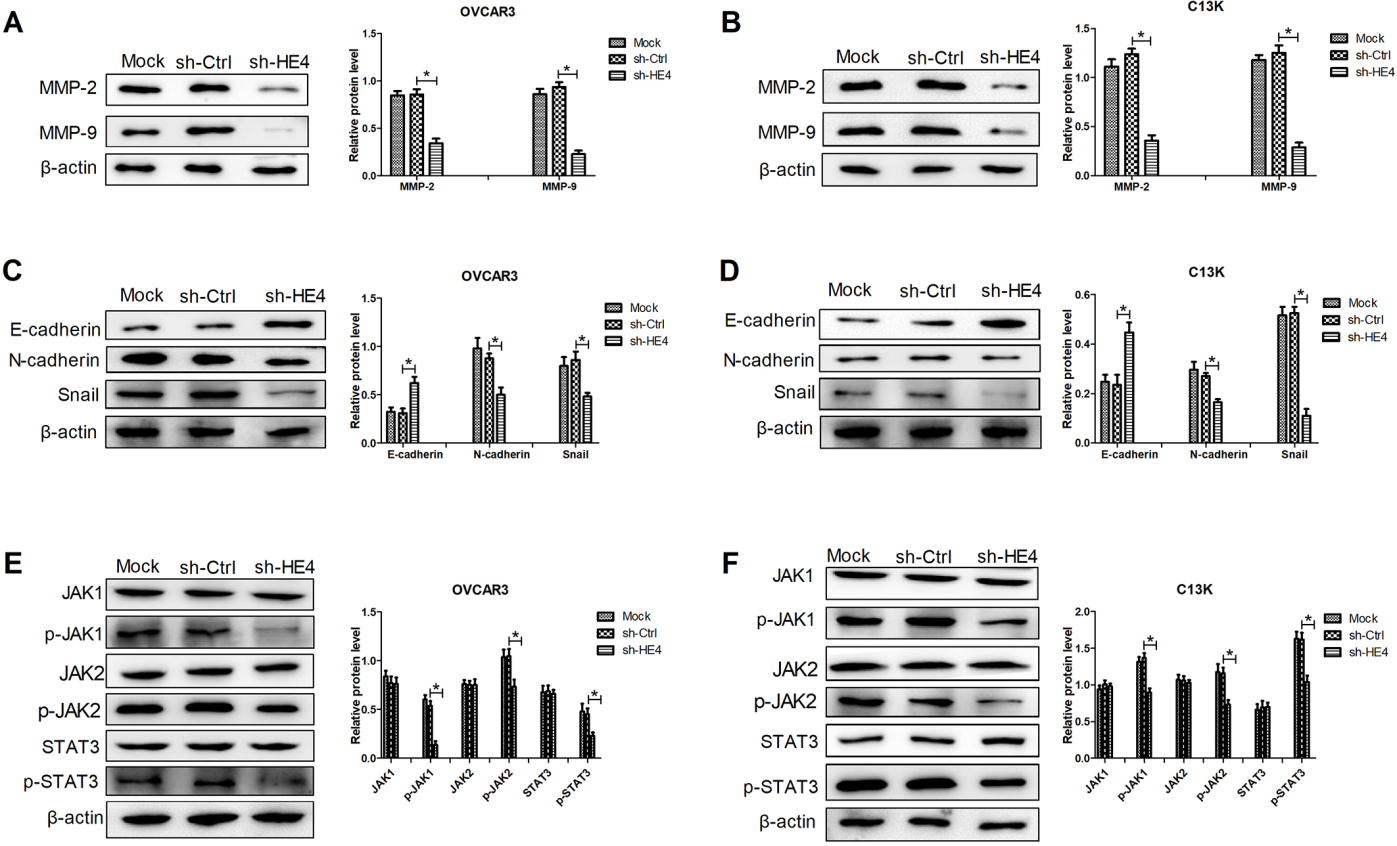
**Downregulation of HE4 inhibited MMPs expression, mesenchymal transition (EMT) and the JAK/STAT3 pathway in ovarian cancer cells.** Western blot analyses of MMP-2 and MMP-9 expression in OVCAR3 (A) and C13K (B) cells transfected with sh-HE4 or sh-Ctrl. Western blot analyses of EMT-related proteins (E-cadherin, N-cadherin and Snail) in the transfected OVCAR3 (C) and C13K (D) cells. Western blot analyses of the expression of JAK1, p-JAK1, JAK2, p-JAK2, STAT3 and p-STAT3 in the transfected OVCAR3 (E) and C13K (F) cells. *P<0.05.

To expound the molecular mechanism by which HE4 played crucial roles in the biological function of OVCAR3 and C13K cells, western blot analyses were conducted. Previous reports have shown that MMPs are associated with angiogenesis, tumor growth and metastasis, which are involved in tumor enhancing effects (James et al., 2018). Our results showed that HE4 knockdown significantly downregulated the expression of MMP-2 and MMP-9 in OVCAR3 and C13K cells (Fig. 5A,B). EMT is reported to boost malignant progression of many cancers (Burk et al., 2008). Therefore, expression of EMT-related proteins (E-cadherin, N-cadherin and Snail) was measured in the transfected OVCAR3 and C13K cells. Our results uncovered that significantly increased levels of E-cadherin and reduced levels of N-cadherin and Snail were observed in cells transfected with sh-HE4 compared with the control cells (Fig. 5C,D). Interestingly, HE4 knockdown significantly inhibited the expression of p-JAK1, p-JAK2, and p-STAT3 in OVCAR3 and C13K cells (Fig. 5E,F). These results implied that HE4 slicing inhibited MMPs expression, EMT and the JAK/STAT3 pathway, which might inhibit the malignant phenotype of ovarian cancer cells.

### Downregulation of HE4 repressed ovarian cancer xenograft tumor growth through inactivating the JAK/STAT3 pathway *in vivo*

**Fig. 6.**
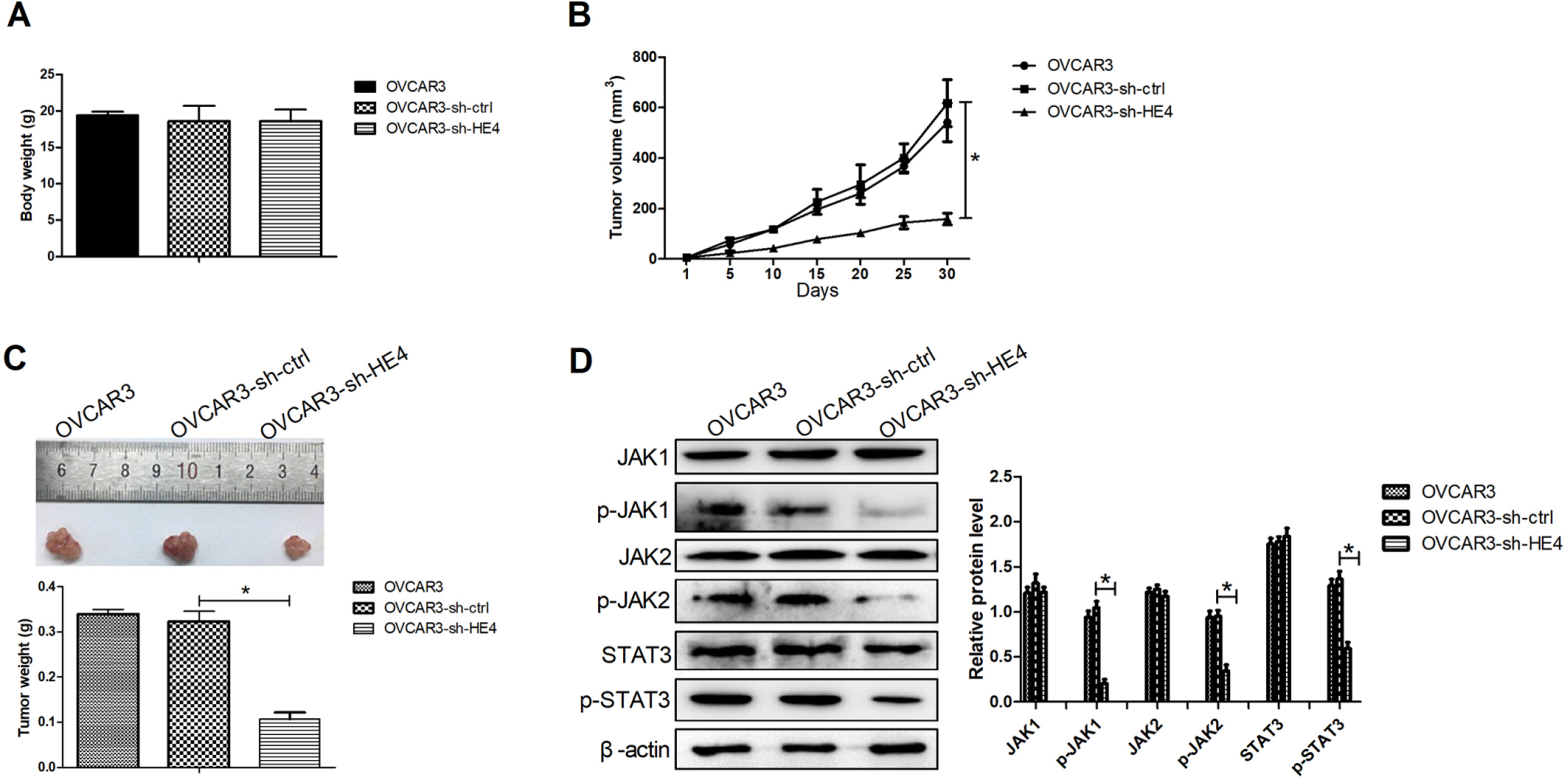
**Downregulation of HE4 repressed ovarian cancer xenograft tumor growth through inactivating the JAK/STAT3 pathway *in vivo.*** Ovarian cancer xenograft mouse models were generated by injection of the transfected OVCAR3 cells subcutaneously into the right dorsal flank of mice (*N*=9). (A) Body weight of mice was measured at 30 days post-injection. (B) The volumes of xenograft tumors were detected by a digital caliper once every 5 days for 30 days. (C) At 30 days post-injection, all mice were euthanized. The xenograft tissues were collected, imaged and weighed. (D) Western blot assays were applied to evaluate the expression of JAK1, p-JAK1, JAK2, p-JAK2, STAT3 and p-STAT3 in xenograft tissues. *P<0.05.

To confirm the relevance of HE4 expression in ovarian cancer cells *in vivo*, ovarian cancer xenograft mouse models were constructed by the injection of the transfected OVCAR3 cells subcutaneously into the right dorsal flank of mice. HE4 downregulation had no effect on body weight of mice (Fig. 6A). However, the size of xenograft tumor was significantly inhibited in the OVCAR3 cells transfected with sh-HE4 compared with the control group (Fig. 6B). Moreover, HE4 knockdown also significantly reduced the xenograft tumor weight compared with the control group (Fig. 6C). Additionally, the results from western blot assays revealed that knockdown of HE4 obviously inhibited expression of p-JAK1, p-JAK2 and p-STAT3 in xenograft tumor tissues. These data suggested that HE4 knockdown dampened ovarian cancer xenograft tumor growth through inactivating the JAK/STAT3 pathway *in vivo*.

## DISCUSSION

Ovarian cancer is reported to be the most lethal gynecological cancer, and its mortality rate in advanced patients has been up to 75% (Zhang et al., 2014). Ovarian cancer is a neoplasm that features rapid growth and disseminated metastasis (Zhang et al., 2018). Because there are no explicit symptoms concerned with the disease in the early stage, it is difficult to diagnose early. Prior studies have pointed out that metastasis and invasion of ovarian cancer at an early stage are the main causes for its high mortality and poor prognosis (Zhang et al., 2014). Therefore, it is critical to elucidate the occurrence of malignant biological properties and molecular mechanisms underlying ovarian cancer to improve patient prognosis.

Schummer et al. first found that HE4 is expressed primarily in some ovarian cancers using comparative hybridization of cDNA arrays (Schummer et al., 1999). Recently, a growing number of researchers have focused on its clinical application as a biomarker for ovarian cancer in early-stage diagnosis. Molina and his colleagues have evaluated the sensitivity of HE4 and CA125 as tumor markers in ovarian cancer at an early stage and HE4 has served as a marker with a higher sensitivity, specificity and efficiency than CA125 and ROMA algorithm (Molina et al., 2011). A strong relationship between serum HE4 and the prognosis of epithelial ovarian cancer patients has been reported in a previous study that uncovers that serum HE4 can provide additional prognostic information for ovarian cancer patients (Kong et al., 2012). Recently, a study has reported that the mortality risk was significantly increased in ovarian cancer tissue with an H-score>4 (HE4 expression), which suggests that expression of HE4 is related to a worse prognosis for patients (Lee et al., 2017). Liao et al. have reported that patients with ovarian cancer have HE4-positive urine, which is similar to serum samples, and detection of HE4 urine level can complement serum detection of ovarian cancer (Liao et al., 2015). Consistent with those results, our study showed that HE4 expression levels in tissue, serum and urine of ovarian cancer patients were significantly upregulated. According to the above results, HE4 functions as one of the important tumor markers for ovarian cancer. However, little is known about the function and mechanism of HE4 in the malignant biological behaviors of ovarian cancer.

It is well known that ovarian cancer features rapid growth of abnormal cells in the ovaries. It has been reported that forced expression of HE4 boosts cell proliferation, cell invasion, metastasis and tumor growth capability, expediting the malignant phenotypes in ovarian cancer (Li et al., 2013; Zhu et al., 2016). Moreover, as mentioned in a recently reported literature review, metastasis is a complicated process where many vital interactions between cancer cells and lots of stromal components are involved in the tumor microenvironment, significantly affecting various aspects of the metastatic cascade in disseminating cancer cells (Motohara et al., 2018). A previous report has shown that knockdown of HE4 has been found to dampen the capabilities of cell proliferation, motility and invasion in ovarian cancer cells (Zhu et al., 2013). Consistent with the previous study, our results also showed that HE4 downregulation significantly inhibited the cell proliferation, invasion and migration of ovarian cancer cells *in vitro*. Moreover, our results exhibited that HE4 downregulation could induce the apoptosis in ovarian cancer cells by enhancing the caspase-3 activity and cleaved caspase-3 expression.

There is some evidence to emphasize that EMT-related reversible plasticity and the reverse process – mesenchymal to epithelial – are important for final developmental cellular differentiation and for clonal outgrowth at metastatic sites in cancers (De Craene and Berx, 2013). The aberrant induction of EMT plays a crucial role in invasion and metastasis of cancers, including ovarian cancer (De Craene and Berx, 2013; Little et al., 2016). Therefore, we detected alterations in the EMT-related proteins’ (E-cadherin, N-cadherin and Snail) expression and interestingly, found that HE4 knockdown caused the significantly increased E-cadherin and reduced N-cadherin and Snail levels in ovarian cancer. According to a previous study, during the EMT process cells gain invasive and migratory behaviors, and accompanying that, vimentin and some MMPs are overexpressed in ovarian cancer (Vergara et al., 2010). In our study, we found that HE4 knockdown inhibited the expression of MMP-2 and MMP-9 in ovarian cancer cells. The importance of the JAK/STAT3 pathway in tumorigenesis and progression of cancer has been emphasized (Jo et al., 2012; Reng-Yun et al., 2014). Liu et al. have found that in lung cancer, the activation of JAK/STAT3 pathway enhances TGF-β-induced EMT and cancer cell migration and invasion through boosting p-Smad3 and Snail expression (Liu et al., 2014). Our results showed that HE4 knockdown dampened the activation of JAK/STAT3 pathway in ovarian cancer *in vitro* and *in vivo*. Given the above results, we concluded that knockdown of HE4 suppressed aggressive cell growth and malignant progression of ovarian cancer by inhibiting the JAK/STAT3 pathway.

## CONCLUSION

In conclusion, our findings suggested that HE4 knockdown mediates the reduced cell proliferation, invasion, migration and tumor growth as well as increased apoptosis through inactivation of the JAK/STAT3 pathway, which provides us a better understanding the function and mechanisms of HE4 in malignant progression of ovarian cancer and might promote to develop a new therapeutic and promising option for patients with ovarian cancer.

## MATERIALS AND METHODS

### Human tissue, serum and urine samples

Benign, tumor and normal tissue samples were collected from 50 patients with benign ovarian cancer, 51 patients with ovarian cancer and five healthy volunteers, respectively. Serum and urine samples were gained from 20 healthy volunteers, 50 patients with benign ovarian cancer, and 51 patients with ovarian cancer. All cases come from the Third Affiliated Hospital of Zhengzhou University. Following centrifugation, the samples were stored at –80°C for qRT-PCR and ELISA assays. All procedures were approved by the Ethics Committee of the First Affiliated Hospital of Zhengzhou University and written informed consent was obtained from all recipients enrolled.

### Cell culture

Human normal ovarian epithelial cell line HOSE and ovarian cancer cell line OVCAR3 were purchased from ATCC (Manassas, VA, USA). C13K cells were gifts from Dr Rakesh GOEL at the Ottawa Regional Cancer Center, Ottawa, Canada. HOSE, OVCAR3 and C13K cells were cultured in RPMI-1640 (Invitrogen, Carlsbad, CA, USA) medium containing 10% fetal bovine serum (FBS; Invitrogen) in a humidified incubator with 5% CO_2_ at 37°C. HEK293T cells were gained from ATCC and grown in Dulbecco's modified Eagle's medium (DMEM; Sigma-Aldrich, St Louis, MO, USA) with 10% FBS under standard conditions.

### Quantitative reverse-transcriptase polymerase chain reaction (qRT-PCR)

Total RNAs were extracted from tissue and cell samples using Trizol agent (Invitrogen) and cDNA was synthesized using PrimeScript 1st Strand cDNA Synthesis Kit (Takara, Dalian, China). Then, qRT-PCR assays were performed on Applied Biosystems 7500 Real-Time PCR using SYBR Premix Ex Taq™ (Takara). All procedures were following the manufacturer's instructions. GAPDH was used as an internal control for mRNA quantification. The relative expression levels of HE4 mRNA were calculated by the 2^−ΔΔCT^ method. The following sets of primers were used in this study: HE4 forward: 5′-CCGACAACCTCAAGTGCTG-3′; HE4 reverse: 5′-CGAGCTGGGGAAAGTTAATG-3′; GAPDH forward: 5′-TGAGAGGGAAATC GTGCGTGAC-3′; GAPDH reverse: 5′-AAGAAGGAAGGCTGGAAAAGAG-3′.

### Enzyme-linked immunosorbent assays (ELISA)

The frozen serum and urinary samples were thawed slowly at 4°C. Half an hour before experiments, the samples remained at room temperature. The levels of HE4 were determined by a HE4-specific sandwich ELISA assays following the manufacturer's protocol (KA&M, Shanghai, China). The specific procedure was modified according to previously reported methods (Iwahori et al., 2012). The absorbance at 450 nm was read on an MK3 microplate reader (Waltham, MA, USA).

### Western blot assays

The total proteins from tissue and cell samples were washed three times with PBS. Then the samples were lysed in RIPA lysis buffer for 30 min. The protein lysates were separated by 10% SDS-PAGE. The specific proteins were transferred on PVDF membranes (Millipore, MA, USA). The membranes were blocked with 5% non-fat milk in TBST for 30 min and then washed twice with TBST. The specific proteins were incubated with the different primary antibodies overnight at 4°C. The used primary antibodies were as follows: JAK1 (Ab-1022), p-JAK1 (SAB4300123), JAK2 (Ab-570), p-JAK2 (SAB4301238), p-STAT3 (SAB4300034), Ki67 (SAB4501880) and STAT3 (SAB4502871) (Sigma-Aldrich), cleaved caspase-3 (9654), matrix metalloproteinases [MMP-2 (40994) and MMP-9 (15561)], E-cadherin (14472), N-cadherin (4068), Snail (3879) and β-actin (8457) (Cell Signaling Technology, Beverly, MA, USA). The membranes were washed twice and then incubated with HRP-linked secondary antibody (7075; Cell Signaling Technology) for 1–2 h. Protein signals were visualized using enhanced chemiluminescence reagents (ECL; GE Healthcare, Waukesha, WI, USA).

### Plasmid construction and stable transfection

Two short hairpin RNAs (shRNAs) targeting HE4 (sh-HE4-1 and sh-HE4-2) were designed according to the coding sequences of the HE4 (GenBank Accession: NC_000020.11) and synthesized. The sequences coding for the sense strand of shRNAs were as followed: sh-HE4-1-F: GCTCCAGGCTGACCAGATTCAAGACGTCTGGTCAGCCTGGAGCTCG; sh-HE4-2-F: CTGCCCCCAGGTGAACATTATTCAAGACGTAATGTTCACCTGGGGGCAG. The two shRNAs were inserted downstream of a human U6 promoter in the pLenti-hU6-EF1-GFP-Puro lentiviral vector according to the previous reported methods (Qin et al., 2003). To construct the shRNA-expressing lentiviral vectors, the shRNA expression cassettes were subcloned into the pLenti-hU6-EF1-GFP-Puro plasmid between the AgeI and EcoRI sites.

The recombinant plasmids expressing shRNAs (sh-HE4-1 and sh-HE4-2) were constructed by calcium phosphate mediated transient transfection of three plasmids including auxiliary plasmids (pCMV-Δ8.2 and pCMV-VSV-G) and the specific plasmid into HEK293T cells. After transfection for 48 h, the virus particles were harvested and filtered. The virus particles carrying sh-HE4-1 and sh-HE4-2 were used to infect OVCAR3 and C13K cells to gain the stable cells. The efficiency of HE4 knockdown was confirmed by qRT-PCR and western blot assays.

### 3-[4, 5dimethylthiazol-2-yl]-2, 5-diphenyltetrazoliumbromide (MTT) assays

The transfected cells were plated on 96-well plates at a density of 1×10^3^ cells/well and five duplicate wells were set. The cells were cultured at 37°C in 5% CO_2_ for 0, 1, 2, and 3 days. Then, 20 µl of 0.5% MTT reagent (Sigma-Aldrich) was added into each well at the indicated times and incubated at 37°C for another 4 h. MTT was discarded and 100 µl of dimethyl sulfoxide (DMSO; Sigma-Aldrich) was added into wells. The plates were slowly agitated for 10 min. Finally, the OD values were read on Bio-Tek Microplate reader EL 800 (Bio-Tek Instruments, Inc.).

### Flow cytometry analyses

The transfected cells cultured for 48 h were trypsinized with 0.25% Tyrisin (Invitrogen) and resuspended in RPMI-1640 media with a final concentration of 1×10^6^ cells/ml. Before flow cytometry analyses, the cells were washed three times with cold PBS and resuspended in 500 µl of binding buffer. 10 μ1 of 7-AAD (BD Biosciences Pharmingen, San Diego, CA, USA) and 5 μl of Annexin V-PE (BD Biosciences Pharmingen) were added into the cell suspension and incubated for 10–15 min on ice. The apoptotic cells were analyzed in FACScan flow cytometers (BD Bioscience).

### Detection of caspase-3 activity in ovarian cancer cells

The transfected cells were seeded in six-well plates and cultured for 48 h. Then, the total proteins were extracted in culture supernatants and the activity of caspase-3 was measured by ApoTox-Glo™ Triplex assay kit ELISA (Promega, WI, USA) according to the manufacturer's instructions. OD values were read using an ELISA MK3 microplate reader (Waltham, MA, USA).

### Transwell assays

Prior to experiments, the transwell chambers (Corning, Acton, Massachusetts) with 8 μM pore membranes were coated with Matrigel (BD Biosciences). The transfected cells were collected in serum-free RPMI-1640 media with a final concentration of 4.5×10^5^ cells/ml and then 300 μl of cells were plated on the top chambers. The medium containing with 20% serum (for all other cells) served as a chemoattractant in the lower chamber. The cells were allowed to invade through Matrigel for 48 h at 37°C. The cells invaded to the lower surface of the membrane were fixed with 5% glutaraldehyde, and stained with 0.2% Crystal Violet. Finally, the stained cells were observed under an optical microscope and counted in five random fields.

### Wound healing assays

Cell migration capability was detected by the wound healing assays. In brief, the transfected cells were plated on six-well plates and when the cells were grown to 90% confluence, a scratch was made on the cells using a 200 μl sterile pipette tip. The cells were washed with PBS to discard the detached cells and debris and then were instantly imaged. After another 24 h incubation in serum-reduced medium supplemented with 2% FBS, the cells were imaged.

### Xenograft tumor assays

300 μl of the transfected OVCAR3 cells (2×10^5^ cells/ml) were injected subcutaneously into the right dorsal flank of 8-week-old female Balb/c nude mice (*N*=9). Volumes of xenograft tumors were determined using a digital caliper once every 5 days for 30 days. And xenograft tumor volumes were calculated by length×width^2^/2. After 30 days, the mice were euthanized and xenograft tumor tissues were collected, imaged and weighed. Then, xenograft tumor tissues were fixed with 4% paraformaldehyde and stored at −80°C for western blot assays. All procedures were approved by the Ethics Committee of the First Affiliated Hospital of Zhengzhou University.

### Statistical analysis

Data were expressed as means±s.d. and analyzed using the SPSS 19.0 software. Student’s *t-*test was applied for comparing differences between two groups and one-way analysis of variance (ANOVA) was used for comparison of differences among more than two groups. *P*<0.05 was considered statistically significant.
